# An Evidence-based Look at the Effects of Diet on Health

**DOI:** 10.7759/cureus.4715

**Published:** 2019-05-22

**Authors:** Sean Kandel

**Affiliations:** 1 Internal Medicine, University of Connecticut, New Britain, USA

**Keywords:** diet, evidence based, cardiovascular disease, stroke, disease risk

## Abstract

Diet is a daily activity that has a dramatic impact on health. There is much confusion in society, including among medical professionals, about what constitutes a healthy diet. Many reviews focus on one aspect of healthy dietary practices, but few synthesize this data to form more comprehensive recommendations. This article will critically review and holistically synthesize the data on diet with firm morbidity and mortality endpoints derived from three key, high quality studies, which are further supported with several additional articles. Specific recommendations of types and quantities of food to reduce the risk of heart disease and stroke will be provided. The effect of these diets on cancer and mood disorders as covered in the articles reviewed will also be discussed.

## Introduction and background

Why do we care about diet?

Cardiovascular disease (CVD) is the number one cause of death in the United States [1]. Obesity and type 2 diabetes, both directly diet modifiable, are two of the largest contributors to CVD. Additionally, cancer and mood disorders both also have significant impacts on morbidity and mortality. What we eat may allow us to powerfully intervene on these issues, which are the largest health issues affecting our country. 

Article selection methods

A keyword search was performed in the PubMed database through July 2018 using permutations of the phrases: “diet randomized controlled trial,” “diet morbidity mortality,” and “fish coronary artery disease.” Fish was specifically searched for as it has been identified as possibly being cardioprotective, as noted by the American Heart Association. Only randomized controlled trials, large, carefully controlled observational studies, or meta analyses looking at firm morbidity/mortality endpoints were considered. Three such representative articles will be reviewed in depth here, with many supporting articles also referenced. Although some data related to cancer and mood disorders is discussed, the primary focus of this review is the effect of diet on coronary artery disease (CAD) and stroke.

What we already knew about diet and its effects on health

Most of the data we have about diets are from observational studies. Some of these studies are outstanding, but nonetheless are limited by the biases that any observational study can have (i.e. confounding variables and correlation without demonstration of causation). Despite this, several massive studies have been replicated and statistically controlled to afford some general trends worth noting. Studies looking at up to 2.88 million patients have reproducibly shown that being at extremes of weight (either morbidly obese or underweight) leads to an increased risk of death, and being overweight or obese leads to an increased risk of death from diabetes or kidney disease [2-3]. Abdominal obesity has been shown to increase coronary artery plaque deposition on computed tomography (CT) imaging [4]. Thus, being a healthy body weight is the first step in reducing cardiovascular risk. The Dietary Approaches to Stop Hypertension (DASH) diet randomized control trial showed that a diet that favors vegetables, fruits, low fat dairy, and minimizes red meats, fats, oils, and snacks/sweets (processed foods with high sugar/simple carbohydrates) leads to a slightly lower blood pressure (about a 5.5 mm Hg and a 3 mm Hg lower systolic and diastolic, respectively) when applied to hypertensive patients [5]. The causative role of diabetes on (CAD) and kidney disease has been well documented [6]. Notably, type 2 diabetes has a dramatic role in morbidity and mortality, and despite some people having a genetic predisposition is ultimately diet induced. It can be prevented by eating a healthy diet, and even after its development can usually be completely reversed with diet and exercise, even in later stages [7-8].

Does low cholesterol correlate with a lower risk of cardiovascular disease?

The foundational studies looking at cholesterol and its effects on health, based on the Framingham study, did not definitely show that the cholesterol levels predicted CVD risk in the general population. The study only achieved statistical significance for total cholesterol in certain age groups (men over age 65 years and women between the ages of 50-79 years). The study did show that the total cholesterol to high density lipoprotein (HDL) ratio seemed to be more predictive of cardiovascular events than the absolute cholesterol numbers [9]. Subsequent to this, 19 studies looking at low density lipoprotein (LDL) cholesterol showed no association or an inverse association between LDL levels and CVD in people over the age of 60 years [10]. Low HDL did seem to have a correlation with CAD in these studies. Notably, in a review of 115,000 patients who had a myocardial infarction, patients with lower LDLs had higher mortalities post myocardial infarction [11]. This study is referred to as “The Lipid Paradox.” In addition, despite eating very high levels of saturated fat and cholesterol, the French have very low rates of CVD [12] - this is referred to as “The French Paradox.” 

If we are to follow the logic that the higher the cholesterol level, the more plaque deposits in the coronary arteries, and thus the more cardiac ischemia/infarctions occur, you would expect that as a person ages and continues to eat unhealthy food that the amount of plaque deposition and ischemia/infarction would also increase. However, this is not the pattern seen in the original Framingham study publication. We also have “The Lipid Paradox,” “The French Paradox,” and a meta-analysis of 19 studies showing no or an inverse correlation of LDL with risk of CVD. LDL cholesterol levels likely do not correlate with the development of CAD as well as was once believed.

Effects of diet are hard to study

The above discussion of the lack of correlation of LDL cholesterol levels with the development of CAD is important to understand why the articles discussed in this review were chosen and why others were excluded. The effects of diet are cumulative and take decades to manifest. However, most researchers want answers much sooner than this, and thus turn to surrogates to measure dietary effects on health, with the most common surrogate being cholesterol. However, as discussed above, cholesterol levels may not reliably predict CAD. Studies that evaluate the effects of diet based on firm endpoints, such as a diagnosis of myocardial infarction, stroke, cancer, or mood disorder, are the most reliable data from which to draw conclusions. This literature is the focus of the remainder of this article.

## Review

The Mediterranean diet

Background

Estruch and his colleagues have produced a large randomized controlled trial looking at the effects of diet on health with hard morbidity and mortality endpoints [13]. Their study was recently retracted and republished after they discovered that 1588 of the 7400 participants really weren’t randomized. The resubmitted work includes statistical modeling which validates their initial results despite the non-randomized group. 

Design

The study design involved a parallel group randomized control trial in Spain with three groups: a Mediterranean diet group supplemented with extra-virgin olive oil (1L per week, at least 4 tablespoons per day), a Mediterranean diet group supplemented with nuts (30 g per day: 15 g of walnuts, 7.5 g of hazelnuts, and 7.5 g of almonds), and a low fat control group. Dietary training sessions were done at baseline and quarterly, and 14 item dietary screeners to assess adherence were also done at baseline and quarterly. The study spanned just over seven years from October 1, 2003 to December 1, 2010.

Results

The primary endpoint was a major cardiovascular event, which was a composite made up of stroke, myocardial infarction, and death from “cardiovascular causes.” The primary endpoint achieved statistical significance in the two Mediterranean diet groups, thus the authors concluded that a Mediterranean diet results in a reduction in all major cardiovascular events. However, in reviewing the subgroup analysis of secondary endpoints, only stroke achieved statistical significance, with the highest statistical significance being in the nut supplemented group. Thus, although the primary endpoint achieved statistical significance, it was entirely powered by a reduction in stroke. In addition, although the intention was to test the Mediterranean diet against a low fat diet, review of the dietary adherence data shows that all three groups, including the control group, scored the same for adherence to the Mediterranean diet. Thus, all three groups were eating the Mediterranean diet. As a result, this study is really comparing the effect of nut or olive oil supplementation on top of the Mediterranean diet compared to the Mediterranean diet by itself. In the re-published version of their article the authors have changed the wording of their conclusions to more closely reflect this. 

Limitations

The study has several limitations. As mentioned above, approximately 20% of participants were not actually randomized and required post study statistical analysis to try to control for this. Also, the study design was changed midway through from 9000 people over four years to 7400 patients over six years, which could have affected the data analysis. Patients in this study were aged 55-80 years and were higher risk patients (with diabetes or multiple other cardiovascular risk factors) which may limit the study’s generalizability to younger, healthier populations. Also, almost all participants were Caucasian, which raises the question of the effects of this diet on other races. Finally, the control group had a higher rate of obesity than the two Mediterranean diet groups, which may have skewed the poor outcomes towards the control group as obesity itself is a risk factor for CAD.

Conclusions

This study shows that eating a significant amount of olive oil or nuts on top of a Mediterranean diet reduced the risk of stroke among high risk Caucasian individuals. 

The China Study

Background

The China study is a compilation of studies related to two large observational studies and related bench research. The primary goal was to assess the effect of varying dietary patterns on the inhabitants of rural China on several health parameters [14-15]. 

Design

The primary studies were observational and done in counties throughout rural China. A total of 6500 adults in 65 counties (with a 50:50 male to female ratio) were involved in the initial study done in 1983-1984. These same 6500 adults were studied again in 1989-1990 with additional participants added to make the second total study population encompass 10,200 participants in 69 counties. Study participants were surveyed for dietary habits (questionnaires and three-day dietary information) and had concurrent laboratory testing (blood, urine, and food samples). Mortality rates for approximately 50 different diseases were reviewed, and dietary, lifestyle, and disease characteristics were analyzed for trends.

Results

CAD mortality rates in China were 4.0 per 100,000 for men and 3.4 per 100,000 for women, compared with 66.8 per 100,000 men and 18.9 per 100,000 women in the United States. These figures represent a remarkable 16.7-fold and 5.6-fold greater mortality rates among US men and women, respectively, than among their Chinese counterparts. The combined CAD mortality rates for both genders in rural China were inversely associated with the frequency of intake of green vegetables (p =0.01). Notably, one of the regions studied that had some of the lowest rates of animal product consumption had no CAD during the entire study period. The authors concluded that a diet comprised of a variety of high-quality plant-based foods yields the lowest disease rates, and that, “there is no evidence of a threshold beyond which further benefits do not accrue with increasing proportions of plant-based foods in the diet.” The authors thus advocate a whole food plant-based diet consistent with a vegan diet.

Limitations

Despite advocating a diet that is consistent with veganism, none of the groups studied were vegan. The closest group to this level of animal product restriction ate approximately 10% of the animal protein consumed by its US counterparts. This is a marked reduction in animal product consumption, but is not the same as no animal products. Without tangible evidence of an outcome related to a given action, we cannot assume that because some of the action is good, more is necessarily better. The authors also state that eating eggs and dairy increases CVD, but these results did not achieve statistical significance in their analysis. A study published by Dehghan and colleagues shows just the opposite of this claim, indicating that higher yogurt and milk consumptions was linked to lower risks of stroke and major CVD [16]. Other limitations include that this study was observational, thus it is subjected to the possible biases of observational studies. The study also looked only at the Chinese, thus whether it translates to other races in unclear. The study population tended to be rural and be physically active (i.e. farming, etc.), which may mean that their activity levels may be different than other populations. Finally, their recommendation of adopting a diet consistent with veganism needs to be balanced with the health risks. It is well known that vegans cannot avoid serious cognitive issues and peripheral neuropathies without artificially supplementing their diets with Vitamin B12 [17-19]. Any diet that cannot be healthy without artificial supplementation needs to be questioned for long-term safety and sustainability.

Conclusions

Focusing on the tangible results from the study, that eating about 10% of the animal products that a typical American eats may lead to dramatically lower rates of CAD in the Chinese, is a powerful and compelling discovery. If we assume the average person eats about three meals per day (21 meals per week), that correlates to eating approximately 2-3 meals per week with animal protein with the rest being vegan.

Fish

There are some data that show that eating fish can decrease the severity of mood disorders, but conflicting data also exist and more research is needed to draw definitive conclusions [20-22]. However, overall the trend seems to be that fish consumption (particularly fatty fish such as salmon) lowers the risk of CAD [23-27]. Although there is some conflicting data, many studies show an inverse relationship between fish consumption and risk of CAD. The prospective study populations in the articles reviewed that support this ranged from 852 to 84,000 patients and were monitored between 6 and 30 years of follow up, with three of these studies following patients for 16 or more years. The CAD protective effects seem to be most prominent after 12 years of fish consumption. Critics of fishs’ role in preventing CAD stem from the original studies in the Greenland Eskimos [28]. These studies were subsequently found to be incorrect, as the low perceived rates of CAD were actually found to be an underreporting error due to the studied societies being rural with inaccurate death reporting. However, the five subsequent studies referenced above suggest that fish consumption is beneficial in preventing from CAD. 

The ideal serving of fish is likely around three 4 ounce servings per week; although more may have a small additional cardioprotective benefit, this needs to be weighed against the increased mercury consumption that might occur and its potential adverse neurological effects. This is consistent with the American Heart Association recommendations [29].

What about pescatarians? The Adventist Health Study 2

Background

The China Study suggests that being overall vegan with a few servings of animal products per week may confer the greatest protection against cancer and cardiac disease. Fish consumption is likely cardioprotective. If these two findings combined would confer further health benefits was evaluated in the Adventist Health Study 2.

Design

The Adventist Health study is an observational study that looked at the effects of many different types of diets on CVD and cancer [30]. It involved about 73,000 people over approximately a five-year period. Participants were broken into groups, including non-vegetarian, “semi-vegetarian” (< 1 serving of meat per week), pescovegetarians (eating only fish as their meat), lacto-ovo vegetarians (typical vegetarians that eat eggs and dairy animal products, but not the animal meat itself), and vegans (vegetarians that eat no animal products whatsoever including no meat, eggs, or dairy).

Results

A trend towards reduction in death from all cause mortality, ischemic heart disease, CVD and cancer was seen in almost all categories by the vegetarian groups. It was largely powered by statistically significant reductions in risk of all cause and ischemic heart disease by pescovegetarians in both sexes, along with reductions in “other” causes of death in both sexes by vegans and pescovegetarians. The most statistically significant reductions were in ischemic heart disease among male vegans (55% reduction) and female pescovegetarians (49% reduction). Only pescovegetarians obtained statistically significant reductions in women. Male vegans (42% reduction), pescovegetarians (23% reduction), and lacto-ovo vegetarians (34% reduction) also achieved statistically significant reductions in CVD mortality. No groups achieved statistically significant reductions in cancer risk.

Limitations

The authors note that, “Vegetarian groups tended to be older, more highly educated, and more likely to be married, to drink less alcohol, to smoke less, to exercise more, and to be thinner.” Many of these factors could bias the reduction in mortality risk towards the vegetarian groups regardless of the diet that they ate. This observation may be indicative of the fact that people who take the time to monitor their diet may also be more health conscious in general. It is also important to consider the specific characteristics of the group being studied. Seventh Day Adventists are strict in their observance of a Sabbath day of non-work, and also are required to refrain from all tobacco, alcohol, or illicit drug use. It is important to keep these restrictions and expectations in mind when interpreting this study, as they may have led to underreporting biases. These study results may also not be as directly extrapolatable to the general population given the restrictions on this population’s lifestyle. 

Conclusions

The Adventist Health Study 2 provides evidence that a vegan inclined diet with a few servings of animal product per week may be better at reducing cardiovascular risk than other diets when studied in Seventh Day Adventists. Within vegetarian subtypes, a pescovegetarian diet was best for women and a vegan diet was best for men for cardiovascular protection. Males also experienced a reduction in cardiovascular events on pescovegetarian and lacto-ovo vegetarian diets.

Glycemic index and load

Glycemic index and load refer to the increase in blood glucose caused by eating a particular food. The lower these numbers, the less that blood sugar levels are increased by a given food. The Harvard School of Public Health website provides the glycemic indexes of many common foods [31]; a more comprehensive list was published in 2008 [32]. Eating low glycemic foods is likely healthier for everyone, but it is particularly important for diabetic patients to prevent dangerously high blood sugar spikes. Larger quantities of low glycemic foods (for example garbanzo beans) can be eaten by diabetics with far less impact on the blood sugar than even small quantities of high glycemic index foods (such as Jell-O). Eating lower glycemic index foods may have many additional health benefits, from reducing the occurrence of symptoms with mood disorders [33-34] and reducing fatigue [34] to reducing the risk of acne [35].

Whole grains

A meta analysis of 64 observational and cohort studies showed that whole grain consumption up to 200 g, including whole grain bread, breakfast cereal, and grain products with bran added, has been shown to reduce CAD, CVD, and total cancer [36]. The results in some cases were in a dose-dependent manner, with higher consumption leading to greater risk reduction. A key limitation of this article is that the specific types of whole grains consumed in the various studies were not always clearly identified. Also, cancer was reported as “total cancer,” without discussion of the specific types. These limitations make it difficult to draw conclusions on whether a specific grain type is responsible for the protective benefits, or what specific cancers may be affected by diet.

So what should we eat?

The above articles have made significant contributions to our understanding of the effects of diet on health, but also have limitations. With this in mind, the above literature suggests that a lacto-ovo-vegetarian inclined diet, with around three servings per week of fish such as wild salmon (ideal) or meat may be the best diet to reduce the risk of CVD and stroke. Some data in the articles reviewed suggested that certain cancers and mood disorders may also be modifiable by diet. The emphasis should be on low glycemic foods in their natural, unprocessed form. Green vegetables and complex carbohydrates such as whole grains should comprise the majority of the diet. Eating 200 g per day of whole grains may be more protective than lesser amounts. Nuts and olive oil are likely protective against stroke when eaten regularly. A handout summarizing these findings that can be given to patients can be found in Appendix A. 

These findings make sense from an evolutionary standpoint. Our hunter gatherer ancestors didn’t have the luxury of supermarkets or restaurants, and thus likely had only a few servings of meat or fish per week when they were able to catch their prey. These meals likely served to provide vitamins and nutrients not readily available in plants, most notably vitamin B12. The majority of their diet thus consisted of plants and fruits that were readily available in their environment, creating a diet similar to that shown to be healthy in our studies.

## Conclusions

Although more randomized controlled trial data are needed for more definitive conclusions, our current literature suggests that a lacto-ovo-vegetarian inclined diet, with around three servings per week of fish such as wild salmon (ideal) or meat may be the best diet to reduce the risk of CVD and stroke. In order for any diet to be effective, it must be sustainable. Patients should be advised to pick a diet closest to that described above that they can maintain for maximum health benefits. A healthy diet can dramatically reduce the risk of future health problems.

## Appendices

Healthy eating handout

Key Ideas

Diet, exercise, avoiding smoking, drinking alcohol in moderation, not doing recreational drugs, managing (not avoiding) stress, and getting 7-9 hours of sleep per night are the most important things you can do to improve your health. What you eat is much more important than how much. Your goal should be a mostly vegetarian diet (plant focused, with some servings of eggs and dairy), with a few servings (approximately 3) per week of fish (best) or meat. The emphasis should be on eating food in its natural, unprocessed form. A simple equation is the most important determinant of your weight: calories in minus calories out. Eating healthy will give you more energy, help you think more clearly, prevent heart disease, strokes, and diabetes, and may improve your mood. Eating healthy is not about eating perfectly all the time, it is about establishing overall healthy habits that you can maintain for life. In general, you should be choosing foods that protect your blood vessels in your heart/brain and prevent your blood sugar from being very high (to prevent diabetes, or possibly reverse it if you have it). The glycemic index and load are useful tools to help approximate how much a particular food will spike your blood sugar. You should aim for most of the food you eat to have a glycemic index around 50 or below. If you exercise frequently (such as 5x a week for 30 min each time), you may require more food around or slightly above the glycemic index of 50 cutoff. Helpful lists of glycemic indexes for common foods can be found by searching Google for either the “Harvard School of Public Health Glycemic Index” (website) or the “Diabetes Care Glycemic Index” (journal article - more detailed). Organic foods without pesticides, antibiotics, and added growth hormones are preferable, although this is not always practical. Avoiding foods with preservatives and artificial ingredients/chemicals is ideal. Wash fruits/vegetables before eating. Foods that are high in omega 3 fatty acids (such as salmon, walnuts, and flaxseed) are anti-inflammatory. Inflammation irritates the inside of blood vessels and makes it easier for a heart attack or stroke to occur. So, foods that contain omega 3 reduce inflammation and thus help prevent heart attacks and strokes. These foods may also help improve your mood and reduce anxiety and depression. You should try to include these foods in your diet. In contrast, foods that are high in omega 6 fatty acids and/or arachidonic acid (such as beef) may cause inflammation and thus may increase your risk of heart attack and stroke. Thus, these foods should be eaten sparingly. 

Foods to Eat and Foods to Avoid

In general, you should eat a mixture of complex (low glycemic) carbohydrates, leafy green vegetables, broccoli, low glycemic index fruit, and a few servings per week of fish (ideal) or other animal meat.

Proteins

Ideal: Salmon (should aim to eat 3x/week as has protective effects for the heart and likely for mood, although contains mercury so should not eat every day). Fish in general (contains mercury so do not eat every day), egg whites, beans (have some carbohydrates in them, but low glycemic and mostly fiber, which slows sugar absorption and prevents sugar spikes), nuts (the nuts with the most data are walnuts, hazelnuts, and almonds - eat a handful or two per day, probably protective against stroke).

Also acceptable: Chicken breast with fat removed, turkey breast with fat removed.

Eat sparingly: Beef, pork

Vegetables

Eat as much as you want, unless on Coumadin/warfarin - then avoid leafy greens. Leafy greens (spinach, kale, cabbage, chard, bok choy, collard greens, etc.), broccoli, cauliflower, zucchini/squash, asparagus, celery, onions, bamboo shoots, eggplant, etc.

Fruits

Low glycemic fruits - eat in moderation: Peaches, pears, apples, strawberries, grapes, oranges, blueberries, tomatoes.

Eat sparingly - very high in sugar: Melons (ex. watermelon, cantaloupe, etc.), dates, bananas.

Complex Carbohydrates

In general, the longer you have to cook something, the less it will spike your blood sugar.

Steel cut oats (best) or rolled oats (ok), pearled barley, brown rice (eat in moderation, contains arsenic), sweet potatoes/yams, pasta cooked al dente (still chewy) can be consumed sparingly (unless you are an athlete with high caloric needs, in which case you can eat more frequently), sprouted grains such as sprouted wheat in Ezekiel bread, quinoa, etc. Breads where the first ingredient is a whole grain are still not ideal, but are better than “enriched wheat” or processed bread.

Eat sparingly: Quick oats, white bread, white rice (caution, contains arsenic), potatoes.

Eat sparingly in general: Candy, cakes, chocolate, pastries, high sugar foods, processed foods (such as lunch meats, have been linked to increased cancer).

Drinks

Ideal: Water is the best thing for you. Non-fat/1% organic milk can be consumed in moderation - there is a conflicting research about whether this increases your risk of heart disease or lowers it. Dairy products may also increase your risk of breast cancer.

Almond/soy milk: Can consider soy - has a higher omega 6 (pro-inflammatory) content, although overall is a low sugar and high protein option.

Sparingly consume/avoid: Juices are very high in sugar - they are not the same thing as eating the whole fruit because they lack the fiber and your body does not have to break them down as much. Regular soda has tons of sugar, diet soda has artificial chemicals which are not optimal for your body’s functioning.

A reasonable goal for the typical person without heart disease or strokes would be to allow 1-2 “cheat meals” per week where they can eat whatever they would like, with the rest of the meals strictly adhering to the healthy diet above. If you find it very difficult to control your cravings, consider making most of your cheat meals extra portions of your favorite healthy diet foods.
